# High-Flow Nasal Oxygen versus Conventional Nasal Cannula in Preventing Hypoxemia in Elderly Patients Undergoing Gastroscopy with Sedation: A Randomized Controlled Trial

**DOI:** 10.7150/ijms.91607

**Published:** 2024-03-25

**Authors:** Xin Yin, Wen Xu, Jianlei Zhang, Mingyue Wang, Zhen Chen, Songbin Liu, Yan Xu, Shaowen Xu, Danian Ji, Jingwen Wang, Weidong Gu

**Affiliations:** 1 Department of Anesthesiology, Huadong Hospital Affiliated to Fudan University, Shanghai, China.; 2 Department of Anesthesiology, People's Hospital of Shigatse City, Tibet, China.; 3 Department of Surgical Intensive Care Unit, Huadong Hospital Affiliated to Fudan University, Shanghai, China.; 4 Department of Gastrointestinal endoscopy, Huadong Hospital Affiliated to Fudan University, Shanghai, China.; 5 Department of Oncology, Huadong Hospital Affiliated to Fudan University, Shanghai, China.

**Keywords:** desaturation, high-flow nasal oxygen, gastroscopy, sedation, elderly

## Abstract

**Background:** We aimed to compare the prevention of hypoxemia using High-flow nasal oxygen (HFNO) or regular nasal tubing (CNC) in elderly patients undergoing gastroscopy with sedation.

**Methods:** This study was a prospective, randomized, controlled trial conducted at a single center. We included elective patients aged 65 and above who were undergoing gastroscopy with sedation. In the intervention group (HFNO), we set the oxygen flow rate to 60 liters per minute with an oxygen fraction (FiO_2_) of 0.6, while in the control group (CNC), it was 6 liters per minute. The primary outcome was the occurrence of hypoxemia (defined as Spo_2_ < 90%).

**Results:** A total of 125 participants were enrolled (HFNO group: n = 63; CNC group: n = 62). The occurrence of hypoxemia was found to be significantly lower in the HFNO group compared to the CNC group (3.2% vs. 22.6%, p = 0.001). Additionally, a significantly shorter duration of low oxygen levels was observed in the HFNO group [0.0 seconds (0.0-13.0)] compared to the CNC group [0.0 seconds (0.0-124.0), p<0.001]. Moreover, a higher minimum Spo2 value was achieved in the HFNO group [99.0% (98.0-100.0) vs. 96.5% (91.0-99.0), p < 0.001], and a shorter recovery time was recorded [0.5 minutes (0.0-0.5) vs. 0.5 minutes (0.0-1.0), p = 0.016] in comparison to the CNC group. There were no differences in terms of comfort level [0 (0-4) vs. 0 (0-5), p = 0.268] between the two groups.

**Conclusions:** The HFNO system was determined to be a safe and highly effective method for oxygen delivery, leading to a reduction in the occurrence of hypoxemia in elderly patients undergoing gastroscopy with sedation. It is recommended that HFNO be considered as the standard approach for management in this population.

## Introduction

Gastrointestinal endoscopy under sedation is a highly effective approach for diagnosing and treating gastrointestinal ailments. It offers notable advantages over conventional endoscopy, chiefly in alleviating pain and minimizing stress reactions. This approach enhances the comfort of medical procedures, bolsters the safety of examinations, and elevates patient satisfaction 1-3. Nevertheless, when standard oxygenation is administered through a nasal cannula, complications during sedation, including cyanosis, dyspnea, and hypoxemia, frequently occur, with an incidence rate ranging from 26% to 85% 4, 5. Furthermore, the aging process presents additional challenges during sedation. Elderly patients are susceptible to severe hypoxemia during gastrointestinal endoscopy under sedation, owing to factors such as respiratory depression, reduced lung compliance, or the presence of multiple chronic conditions. This heightened susceptibility places elderly patients at considerable risk, including potentially life-threatening situations 6-8. Consequently, maintaining adequate oxygenation during endoscopy under sedation is of paramount importance for this demographic.

High-flow nasal oxygen (HFNO) is an innovative oxygen supplementation system that delivers heated and humidified gas with an oxygen fraction (FiO_2_) that spans from 21% to 100% at a high flow rate (up to 90 liters per minute) 9. Its application has gained significant traction in the care of patients in intensive care units with hypoxemic acute respiratory failure, chronic obstructive pulmonary disease, and other conditions 10. Notably, in recent times, many clinical researchers have adopted HFNO during anesthesia to enhance oxygenation and reduce the incidence of hypoxemia 11. Several studies have corroborated the effectiveness of HFNO in diminishing the occurrence of hypoxemia and other adverse events when propofol sedation is employed for endoscopic procedures 12, 13. However, the precise role of HFNO in preventing hypoxemia in elderly patients undergoing gastroscopy with sedation remains uncharted territory. Consequently, we have undertaken this prospective study to compare the efficacy in preventing hypoxemia between HFNO and conventional oxygen delivery in this specific patient population.

## Patients and Methods

### Study design

This study followed a prospective, randomized, and single-center clinical trial design. Approval for the study was granted by the Medical Ethics Committee of Huadong Hospital, affiliated with Fudan University (Approval No. 2023K084). All patients provided written informed consent, and the trial was registered with the Chinese Clinical Trial Registry (ChiCTR2300070097). Our study focused on elderly patients undergoing gastroscopy with sedation at Huadong Hospital, affiliated with Fudan University, and it was designed as a prospective randomized controlled trial. Eligible participants were individuals aged 65 years and above, falling into American Society of Anesthesiologists (ASA) physical status classes I-III. Exclusion criteria encompassed a history of heart failure, severe arrhythmia, unstable angina pectoris, severe valvular heart disease, or acute myocardial infarction within the past 6 months. Additionally, individuals with acute upper respiratory tract infections, other severe respiratory conditions, allergies to propofol or eggs, or those requiring emergency procedures were also excluded. Using computer-generated randomization numbers, patients were allocated randomly in a 1:1 ratio to either the intervention group (HFNO) or the conventional nasal cannula (CNC) group.

### Methods

Demographic data and clinical characteristics were collected preoperatively, including patient age, gender, weight, body mass index (BMI), ASA physical status, comorbidities, smoking history, breath-holding time (BHT), mean arterial pressure (MAP), STOP-Bang score, and Mallampati grade.

In the endoscopy room, we applied electrocardiogram monitors (GE Healthcare Finland 0y, Finland) measuring noninvasive blood pressure, electrocardiogram readings, and pulse oximeter saturation (Spo_2_). Patients were positioned in the left lateral position. A special opaque screen was erected to concealed both the head of patients and the HFNO devices (BMC Medical Co., Ltd, China). To eliminate the impact of equipment noise, another anesthesia staff member controlled the HFNO device during the sedation procedures for both groups. Consequently, the anesthesiologist remained blinded, unable to distinguish which oxygenation method was in use. Patients, however, were not blinded as they could perceive the oxygen flow rate.

Before the sedation protocols were initiated, pre-oxygenation was performed for 3 minutes for both groups. In the HFNO group, an oxygen flow rate of 30 liters per minute was utilized via the HFNO system, delivering an inspired oxygen fraction (FiO2) of 0.6 at a temperature of 31°C. In the CNC group, oxygen was administered through nasal cannula at a rate of 6 liters per minute. Patients were directed to breathe spontaneously. For both groups, the sedation protocols were initiated following 3 minutes of pre-oxygenation. Intravenous sedation was administered to all patients: fentanyl (0.5-2 µg·kg^-1^) and propofol (1-2 mg·kg^-1^) were slowly administered until the eyelash reflex was lost and responsiveness to stimulation ceased. In the HFNO group, once an appropriate depth of sedation was achieved, the oxygen flow rate was raised to 60 liters per minute and maintained until the end of the procedure. After sedation, the gastroscopy procedure was conducted by a skilled gastroenterologist. Starting from hypopharynx to the descending part of duodenum, esophagus, cardia, the greater curvature, gastric body, pylorus, and duodenal bulb were observed in sequence. Any identified lesions were documented, and a random biopsy was obtained from the gastric antrum. In cases where suspected tumoral lesions were observed, 1-3 biopsies were taken from the lesion sites. Additional propofol (0.2-0.5 mg·kg^-1^) was administered if agitation or resistance was exhibited by patients. Vital signs and electrocardiograms were vigilantly monitored. Intraoperative blood pressure and intraoperative heart rate, as defined by values recorded at the 3rd minute following the commencement of the gastroscopy procedure, were monitored. Ephedrine was applied as vasoactive agent to uphold hemodynamic stability in cases where MAP decreased to less than 30% of the baseline value or the heart rate dropped below 50 beats/min. The dosage of ephedrine was determined by anesthesiologist.

In the event of hypoxemia (defined as Spo_2_ < 90%) occurring during gastroscopy, interventions such as the jaw thrust maneuver and an increase in the oxygen flow rate were performed by another member of the anesthesia care team. If hypoxemia persisted despite these measures, 100% oxygen was administered via a face mask. Tracheal intubation and further interventions were initiated if severe hypoxia could not be corrected by mask ventilation.

After the procedure, patients were asked to rate their comfort level using a numerical rating scale (NRS) ranging from 0 to 10 14. All the data observed during above procedure were collected by clinical research assistants and statistically analyzed by an independent researcher.

### Outcomes

The primary outcome was the incidence of hypoxemia, defined as Spo_2_<90%, during the sedation. Secondary objectives included: the number of hypoxemia episodes (recording a new episode if Spo_2_ dropped below 90% for at least 30 seconds after recovering to Spo_2_ ≥ 90%), duration of hypoxemia (defined as the time it took to reach Spo_2_≥90%), minimum value of Spo_2_, recovery time, patient comfort, airway interventions, and postoperative adverse events such as hypotension, vomiting, aspiration, and more.

### Statistical analysis

Sample size calculation was conducted using PASS version 15.0 (NCSS LLC, Kaysville, Utah, USA). Based on the results of our pilot study involving 60 patients (results which were unpublished), the incidence of hypoxemia was 25% in the CNC group and 3.7% in the HFNO group. Consequently, a minimum sample size of 128 cases (64 cases in each group) was determined, assuming a two-tailed α=0.05, 80% power of detection, and a 20% dropout rate.

Statistical analysis was performed using SPSS version 27.0 (IBM Corporation, NY, USA). Descriptive measurement data were presented as mean ± standard deviation or median (inter-quartile range) depending on the normality assumption's validity. Group comparisons were conducted using independent t-tests or Mann-Whitney U tests, as appropriate. Enumeration data were expressed in numbers and percentages. Fisher's exact tests or chi-squared tests were employed for between-group comparisons of enumeration data. The level of significance was set at a two-sided p value < 0.05.

## Results

**Figure 1:**
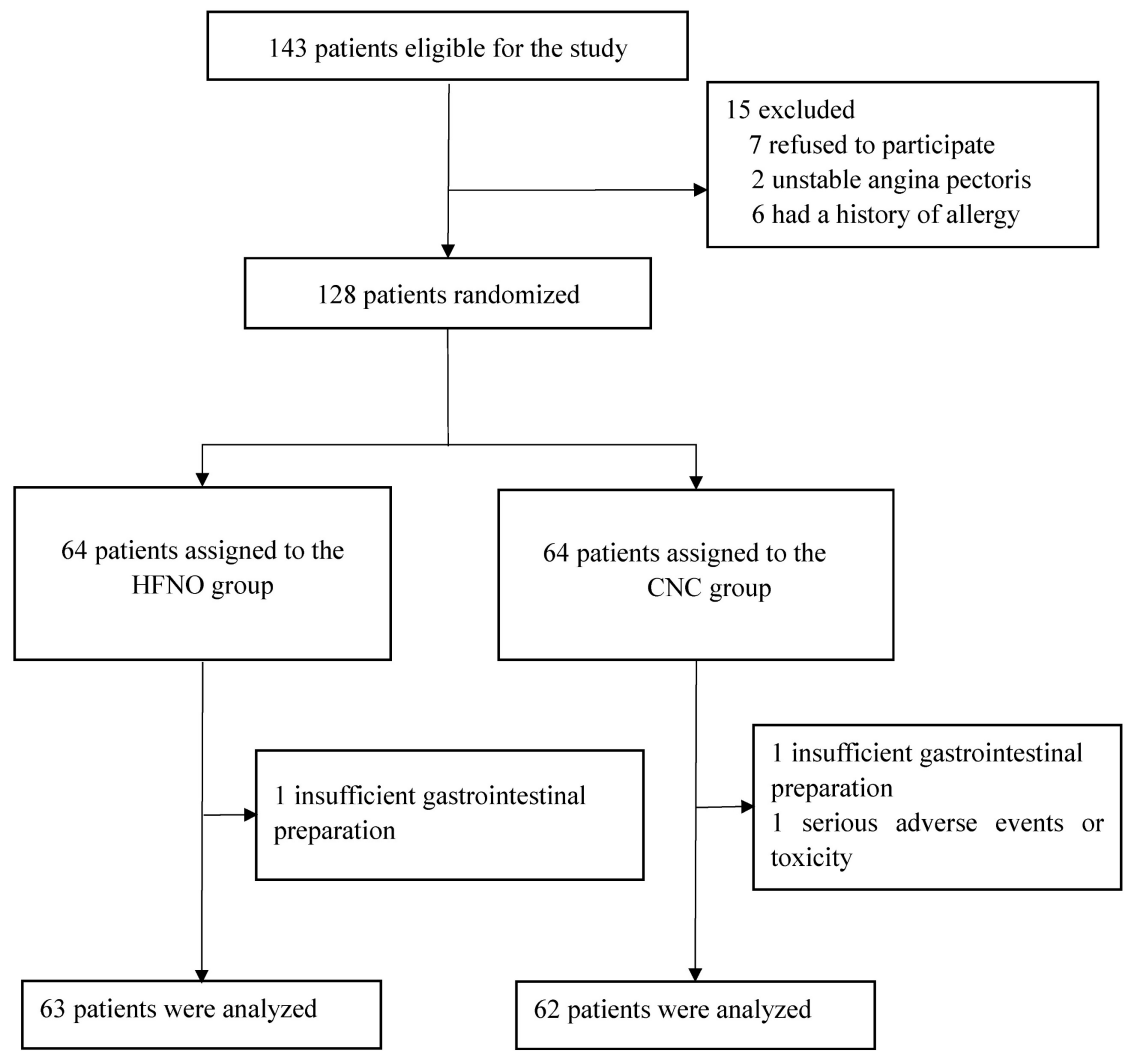
Trial Profile.

Between May 1, 2023, and July 31, 2023, a total of 128 out of 143 screened patients (89.5%) were randomly allocated to either the HFNO group or the CNC group, with 64 patients in each group. Three cases were excluded due to inadequate gastrointestinal preparation and hemodynamic instability, leaving 63 cases in the HFNO group and 62 cases in the CNC group for final analysis. Figure 1 illustrates the trial profile. Baseline demographic and characteristics were evenly distributed between the two groups. There were no statistically significant differences in the propofol dose [1.4 mg/kg (1.2-1.6) vs. 1.3 mg/kg (1.1-1.5), p = 0.378] and fentanyl dose [0.8 μg/kg (0.7-0.9) vs. 0.8 μg/kg (0.7-1.0), p = 0.334] (Table 1).

**Figure 2:**
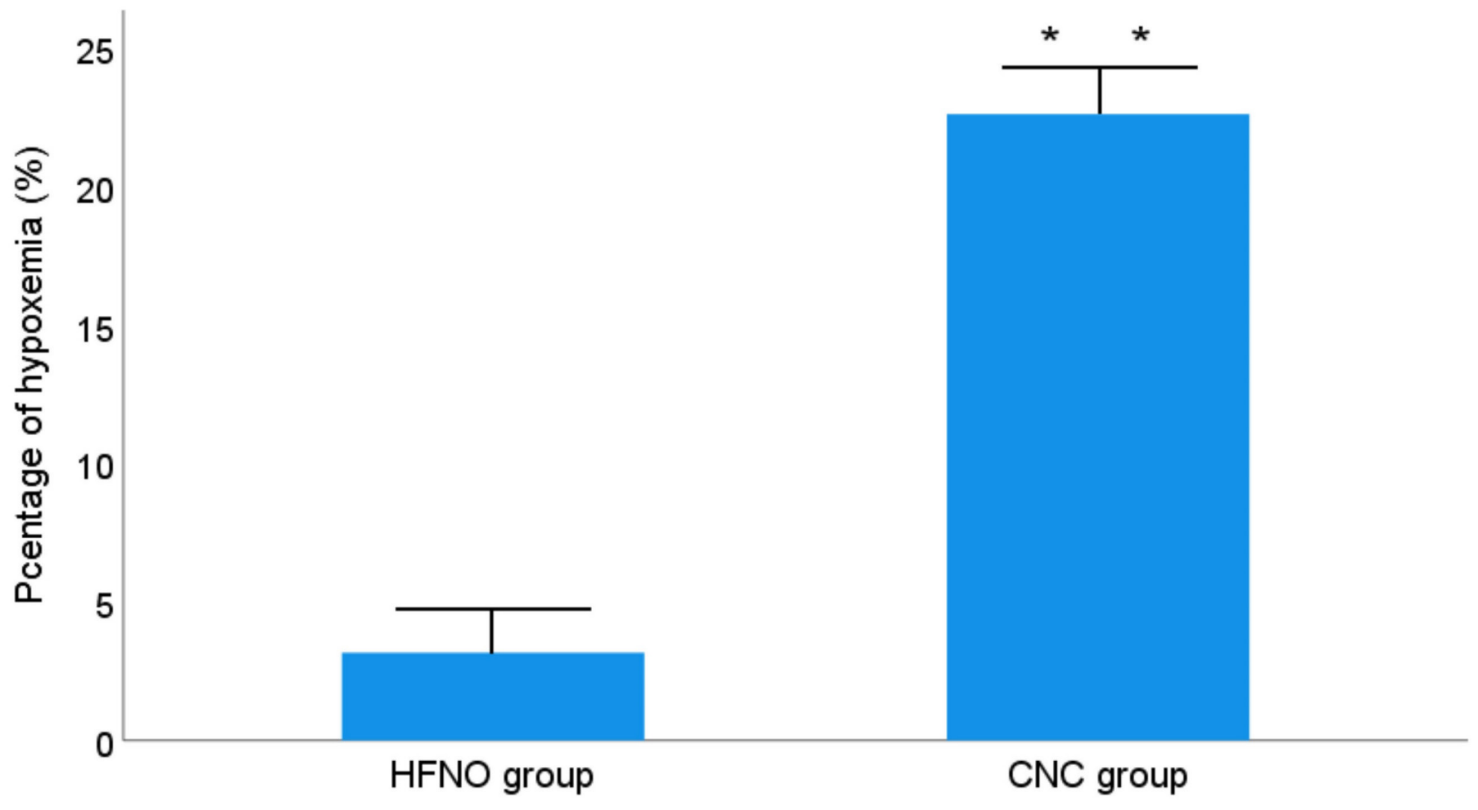
Proportion of patients with desaturation < 90%.

Patients in the HFNO group exhibited a significantly lower incidence of hypoxemia compared to those in the CNC group (3.2% vs. 22.6%, p = 0.001) (Figure 2). Additionally, patients receiving CNC experienced a higher number of hypoxemia episodes [0 (0-1) vs. 0 (0-3), p = 0.001] and longer durations of hypoxemia [0.0 seconds (0.0-13.0) vs. 0.0 seconds (0.0-124.0), p <0.001] compared to those treated with HFNO. The minimum Spo2 value was significantly higher in the HFNO group (99.0% [98.0-100.0] vs. CNC group: 96.5% [91.0-99.0], p < 0.001) (Table 2).

Recovery time was shorter in the HFNO group compared to the CNC group [0.5 minutes (0.0-0.5) vs. 0.5 minutes (0.0-1.0), p = 0.016]. However, no statistically significant differences were observed in sedation time [5.0 minutes (4.0-5.5) vs. 4.5 minutes (3.5-6.0), p = 0.933] and intraoperative MAP (HFNO group: 84.3±12.7 mmHg vs. CNC group: 85.3 ± 13.0 mmHg, p = 0.684) between the two groups (Table 3). Although comfort levels [0 (0-4) vs. 0 (0-5), p = 0.268] and postoperative adverse events [0 (0.0%) vs. 0 (0.0%), p = 1.000] were similar in both groups, the HFNO group required fewer airway interventions performed by anesthesia providers [1 (1.6%) vs. 12 (19.4%), p < 0.001] (Table 3). No patients needed tracheal intubation in our study.

## Discussion

Sedation can enhance patients' tolerance to gastroscopy procedures, thus improving diagnostic accuracy and therapeutic efficacy 15-17. However, complications such as upper airway obstruction during endoscopic intubation and airway collapse induced by medication administration frequently lead to hypoxemia as the most common complication during sedated endoscopy 18. Elderly patients, with reduced respiratory reserve capacity and cardiopulmonary complications, are particularly susceptible to hypoxemia during sedation. Consequently, the administration of sedation in elderly patients poses unique challenges that require heightened attention from anesthesiologists.

HFNO systems can consistently deliver heated and humidified gas with a high FiO_2_ (up to 100%) at substantial gas flow rates (ranging from 30 to 90 L min^-1^). The positive airway pressure generated by HFNO can be adjusted according to the flow rate. Riva et al. reported that HFNO produced flow-dependent positive airway pressures in patients under general anesthesia, with airway pressure increasing nonlinearly with flow rate, suggesting that even small increases in flow rate could result in a substantial rise in pressure 19. Furthermore, the impact of HFNO on airway pressure can translate into increased end-expiratory lung volume. Corley et al. observed a significant simultaneous increase in airway pressure and end-expiratory lung volume in post-cardiac surgical patients receiving HFNO, demonstrating a strong correlation between these two parameters 20. Collectively, these effects not only enhance the efficacy of preoxygenation but also extend the safe apnea duration 20-22. Previous studies have shown that HFNO can prolong the safe apnea time in patients undergoing general anesthesia 23, 24. Nay and colleagues conducted research on patients at high risk of hypoxemia (those with cardiac or respiratory disease, obesity, etc.) undergoing gastrointestinal endoscopy. Their results indicated that HFNO could reduce the incidence of desaturation during sedation compared to conventional nasal cannula 25.

In this prospective randomized clinical trial, we compared the use of the HFNO system (60 L min^-1^) with conventional nasal cannula (6 L min^-1^) in elderly patients undergoing gastroscopy with sedation. Our results demonstrate the advantages of HFNO for these elderly patients. The incidence of hypoxemia in the HFNO group was only 3.2%, markedly lower than the 22.6% observed in the CNC group. Several potential explanations exist for these findings: (1) Approximately one-third of elderly patients experience pharyngeal dysfunction, rendering them susceptible to upper airway obstruction and hypoxemia during anesthesia. However, the positive airway pressure generated by the HFNO system can ameliorate oxygenation in elderly patients with potential pharyngeal dysfunction^23^, ^26^; (2) High gas flow can result in the dead-space washout effect, reducing CO_2_ reinhalation and enhancing ventilation 23, 27, 28; (3) HFNO can provide slight positive end-expiratory pressure (PEEP), positively impacting alveolar recruitment and ventilatory efficiency 19, 29; (4) HFNO surpasses the patient's peak inspiratory flow, reducing the dilution of oxygen by ambient air and maintaining FiO_2_ stability 24, 30, 31; (5) By reducing the work of breathing, HFNO can mitigate the negative effects of sedation on ventilation and the respiratory muscle strength of elderly patients 29, 30. Additionally, we observed fewer episodes of hypoxemia and shorter durations of hypoxemia in the HFNO group. Furthermore, the HFNO group exhibited a higher minimum Spo_2_ value than the CNC group. Collectively, our findings align with previous research indicating that HFNO can augment oxygen supplementation and enhance oxygenation during sedation 6, 31, 32.

We observed that more patients in the CNC group required airway interventions compared to those in the HFNO group. This suggests that HFNO may reduce the workload of anesthesiologists and enhance medical safety. For instance, in one case in the CNC group, severe hypoxemia occurred, and despite immediate interventions like jaw-thrust and mask ventilation, hypoxemia could not be relieved. However, upon switching to HFNO, the patient's SpO_2_ rapidly increased to over 90% and remained stable throughout the procedure. We postulate that HFNO's continuous high-flow oxygen delivery during gastroscopy and airway flushing may facilitate apneic oxygenation 19, 21, 23. Additionally, the heated and humidified gas provided by the HFNO system can alleviate upper airway dryness and reduce the risk of epistaxis, ultimately enhancing patient comfort. This may explain why we did not observe a significant difference in patient comfort between the two groups.

Nonetheless, our study does have several limitations. Firstly, we did not record arterial oxygen saturation (Sao_2_) or other ventilation parameters, such as minute ventilation volume and end-tidal carbon dioxide. This could result in an incomplete assessment and comparison of the physiological effects between the two groups. However, Spo_2_ is strongly correlated with Sao_2_, and to ensure measurement accuracy, we refrained from using vasopressors and only recorded Spo_2_ when the pulse waveforms were stable. Secondly, patients could perceive the degree of oxygen flow from the nasal cannula, making it challenging to blind the patients. This subjective awareness might lead to alterations in their breathing amplitude and frequency, potentially affecting Spo2 results. Furthermore, as we excluded elderly patients with severe respiratory diseases, it remains unclear whether HFNO would benefit this particular patient population. To address this issue, our future research will focus on elderly patients with concurrent respiratory diseases.

## Conclusions

In conclusion, the HFNO system, in comparison to the conventional nasal cannula, proves to be a safe and highly effective method for oxygen supply. It significantly reduces the incidence of hypoxemia in elderly patients undergoing gastroscopy with sedation, thereby enhancing procedural safety.

Based on our findings, we advocate for the widespread adoption of HFNO as the standard approach in managing this patient population.

## Figures and Tables

**Table 1:** Baseline Demographic and Clinical Characteristics

Characteristics	HFNO group (n = 63)		CNC group (n = 62)		*P* value
	n	%	n	%	
Sex					0.940^c^
Male	26	41.3%	26	41.9%	
Female	37	58.7%	36	58.1%	
Age (years)	71.0 (68.0-75.0)		70 (67.0-74.3)		0.546^b^
Weight (kg)	65.5±9.7		63.8±10.8		0.376^a^
BMI (kg/m^2^)	24.2±2.7		23.8±3.2		0.418^a^
ASA					0.892^c^
Ⅰ	22	34.9%	24	38.7%	
Ⅱ	35	55.6%	33	53.2%	
Ⅲ	6	9.5%	5	8.1%	
Complications					
Cardiac disease	19	30.2%	13	21.0%	0.239^c^
Hypertension	34	54.0%	32	51.6%	0.792^c^
Diabetes	7	11.1%	13	21.0%	0.133^c^
Pulmonary disease	11	17.5%	4	6.5%	0.058^c^
Smoking history					0.758^c^
Currently	7	11.1%	8	12.9%	
Previously or never	56	88.9%	54	87.1%	
Breath holding time (sec)	30.0 (29.0-34.0)		32.8 (27.8-42.5)		0.217^b^
Stop-bang grade	3 (2-3)		3 (2-3)		0.476^b^
Mallampati grade					0.895^c^
1	36	58.1%	36	57.1%	
2	22	35.5%	24	38.1%	
3	4	6.5%	3	4.8%	
Baseline MAP (mmHg)	100.5±15.1		97.8±13.2		0.297^a^
Baseline heart rate (bpm)	82.0 (71.0-95.0)		79.0 (72.8-86.0)		0.342^b^
Baseline Spo_2_ (%)	98 (97-98)		98 (97-99)		0.128^b^
Dose of fentanyl (mg)	0.05 (0.05-0.05)		0.05 (0.05-0.05)		0.961^b^
Dose of fentanyl (μg/kg)	0.8 (0.7-0.9)		0.8 (0.7-1.0)		0.334^b^
Dose of propofol (mg)	100.0 (80.0-100.0)		90.0 (70.0-100.0)		0.091^b^
Dose of propofol (mg/kg)	1.4 (1.2-1.6)		1.3 (1.1-1.5)		0.378^b^
Gastroscopic manifestations					0.121^ c^
Gastritis	28	44.4%	36	58.1%	
Peptic ulcer	11	17.5%	6	9.7%	
Polyp	12	19.0%	7	11.3%	
Reflex esophagitis	9	14.3%	6	9.7%	
Postoperative status	3	4.8%	4	6.5%	
Others	0	0%	3	4.8%	

^a^ independent *t*-test. ^b^ Mann-Whitney *U* test. ^c^ *x^2^* or Fisher's exact test. *P*-values < 0.05 were considered statistically significant for all comparisons.

**Table 2:** Respiratory-Related Adverse Events

	HFNO group (n =63)	CNC group (n =62)	*P* value
Hypoxemia (%)	2 (3.2%)	14 (22.6%)	0.001^c^
Duration of hypoxemia [sec, median (min - max)]	0.0 (0.0-13.0)	0.0 (0.0-124.0)	<0.001^b^
Minimum value of Spo_2_ (%)	99.0 (98.0-100.0)	96.5 (91.0-99.0)	<0.001^b^
Hypoxemia episodes [n, median (min - max)]	0 (0-1)	0 (0-3)	0.001^b^

^b^ Mann-Whitney *U* test. ^c^ *x^2^* or Fisher's exact test. *P*-values < 0.05 were considered statistically significant for all comparisons.

**Table 3:** Gastroscopy Procedure Data

	HFNO group (n =63)	CNC group (n =62)	*P* value
MAP (mmHg)	84.3±12.7	85.3±13.0	0.684^a^
Heart rate (bpm)	68.4±9.0	71.1±10.5	0.120^a^
Sedation time (min)^#^	5.0 (4.0-5.5)	4.5 (3.5-6.0)	0.933^b^
Recovery time (min)^*^	0.5 (0.0-0.5)	0.5 (0.0-1.0)	0.016^b^
Comfort level	0 (0-4)	0 (0-5)	0.268^b^
Airway intervention (%)	1 (1.6%)	12 (19.4)	<0.001^c^

^a^ independent *t*-test. ^b^ Mann-Whitney *U* test. ^c^ *x^2^* or Fisher's exact test. *P*-values < 0.05 were considered statistically significant for all comparisons. ^#^ Sedation time: the span of time from the loss of consciousness after administration of sedative to the recovery of consciousness and opening eyes. ^*^ Recovery time: the span of time from scope withdrawal to the recovery of consciousness and opening eyes.
